# Percutaneous Internal Ring Suturing for Inguinal Hernia Repair in Children: Retrospective Cohort of 714 Patients with Minimum 3-Year Follow-Up

**DOI:** 10.3390/medicina60071137

**Published:** 2024-07-15

**Authors:** David Terence Thomas, Sefa Sag, Aybegum Kalyoncu Aycenk, Serkan Tulgar

**Affiliations:** 1 Department of Pediatric Surgery, Maltepe University Faculty of Medicine, Istanbul 34857, Turkey; 2 Department of Pediatric Surgery, Istanbul Sehit Prof. Dr. Ilhan Varank Research and Training Hospital, University of Health Sciences Faculty of Medicine, Istanbul 34764, Turkey; drsefa51@gmail.com; 3 Department of Pediatric Surgery, Ordu University Faculty of Medicine, Ordu 34785, Turkey; aybegumkalyoncu@gmail.com; 4 Department of Anesthesiology and Reanimation, Samsun University Faculty of Medicine, Samsun 55280, Turkey; serkantulgar.md@gmail.com

**Keywords:** inguinal hernia, percutaneous internal ring suturing, long term, follow-up

## Abstract

*Background and Objectives*: Despite numerous described techniques, laparoscopy has yet to replace open surgery as the gold standard for inguinal hernia (IH) repair in children. This may be due to many variables, including the lack of long-term follow-up and concern for increased recurrence. In this study, we present our long-term follow-up data on children undergoing percutaneous internal ring suturing (PIRS) for IH repair. *Materials and Methods*: This retrospective cohort study included children who underwent PIRS for IH between May 2013 and May 2021 at three tertiary care institutions, with at least three years of follow-up. Age at surgery, side of IH, presence of contralateral patent processus vaginalis, surgical and anesthesia time, and complications were noted. Parents were contacted to enquire about long-term complications, such as recurrence. *Results*: Long-term follow-up (average 6.9 ± 2.3 years) was available for 714 patients. For unilateral and bilateral procedures, the average surgical time was 13.6 ± 5.4 and 19.9 ± 3.0, and the average anesthesia time was 27.7 ± 12.9 and 33.9 ± 14.1 min, respectively. Complications were seen in 0.84% of patients and 1.2% of procedures, and recurrence was observed in 0.98% of patients and 0.78% of procedures. *Conclusions*: Our study, with a nearly 7-year follow-up, provides substantial evidence that PIRS is a safe and effective technique for IH repair in children, with low recurrence and complication rates. Despite the study’s retrospective nature and limited sample size, it contributes valuable data supporting the use of PIRS in pediatric IH repair.

## 1. Introduction

Inguinal hernia (IH) is a frequently encountered surgical pathology of childhood, seen in up to 5% of all children. [1]. Although direct IHs are more common in adults, indirect IH caused by a patent processus vaginalis (PPV) is more prevalent in children. Concern for organ incarceration and necrosis necessitates surgical intervention in all children diagnosed with IH [2].

After the first report of a female patient undergoing laparoscopic IH repair in 1997 [3], several intracorporeal and extracorporeal techniques have been described in the literature [4,5,6,7]. As in open herniorrhaphy, the techniques for the laparoscopic repair of IH maintain the fundamental premise of high ligation of the hernia sac, with or without division of the hernia sac. In 2006, Patkowski et al. described “percutaneous internal ring suturing” (PIRS), a laparoscopy-assisted extracorporeal technique. In PIRS, an intravenous cannula needle is used to ligate the patent processus vaginalis at the internal ring level without the necessity for working ports [8,9]. Our surgical and anesthesia-related experiences with PIRS have previously been reported [10,11,12].

Open herniorrhaphy is still considered the preferred surgical procedure owing to excellent success rates and the low occurrence of complications. Although minimally invasive surgical techniques have become the gold standard for the treatment of many surgical pathologies in children, there exists no consensus for the optimum minimally invasive technique for the repair of inguinal hernia in children. This may be the consequence of a multitude of factors, including the absence of data on long-term follow-up and the concern of a potential rise in the recurrence rate when these techniques are utilized.

The aim of this study was to evaluate the long-term outcomes of children who underwent PIRS for IH repair.

## 2. Materials and Methods

This retrospective cohort study was conducted between May and June 2024 and included children undergoing IH repair using the PIRS technique at three tertiary care institutions between May 2013 and May 2021.

**Figure 1:**
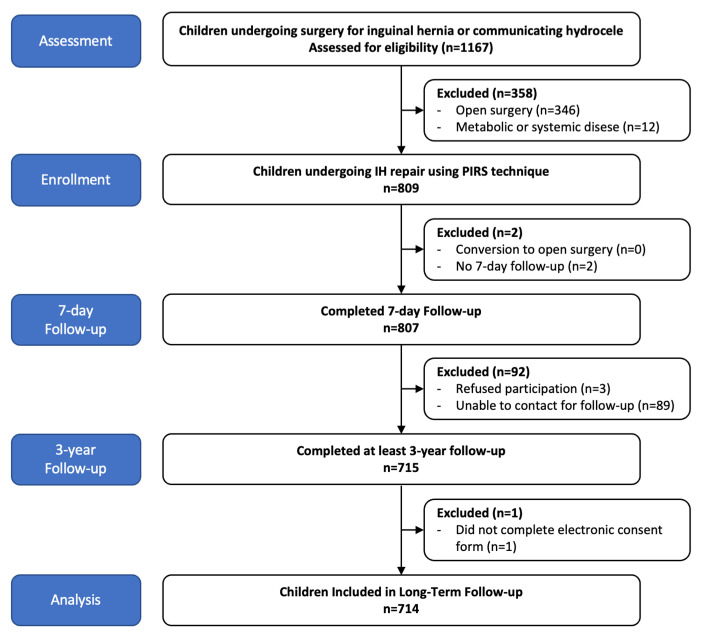
STROBE diagram of participant flow for the study.

Children who underwent PIRS repair for IH that were aged between 0 and 18 years old with at least 3 years of follow-up data were included in the study. Children with metabolic or systemic diseases that could potentially impact the duration of surgery or time under anesthesia were excluded from the study. Figure 1 shows the STROBE diagram of the study.

Children’s age at surgery, presenting side of hernia on first presentation, length of surgery, time under anesthesia, presence of contralateral patent processus vaginalis (CPPV), and intraoperative complications were collected from patient files. The parents/guardians of all patients were contacted by telephone, and the presence of any complications, including recurrence, hydrocele, and testicular atrophy, was questioned and noted.

PIRS was performed as previously described [8,10] in all patients. All procedures were conducted under general anesthesia, and airways were maintained using a laryngeal mask (LMA) or endotracheal intubation (ETT), depending on the anesthesiologists’ preferences. A 3 or 5 mm port was used to insert a 30° telescope, and an insufflation pressure of 8–10 mm Hg was used, providing excellent vision of the surgical field in all patients. Under direct vision, small millimetric incisions were performed immediately above the internal inguinal ring. An 18 G angiocath needle loaded with a 2/0 nonabsorbable monofilament suture loop was passed from one side of the internal ring, entering and exiting the peritoneum several times until the farthest point possible was reached. The loop suture was inserted into the abdominal cavity, and the needle was removed. Thereafter, the needle was reintroduced, this time loaded with a 2/0 nonabsorbable monofilament suture and passed from the opposite side of the initial loop suture placement to the farthest opposite side. The suture was passed through the loop, and the loop was withdrawn, catching the second suture that was then tied subcutaneously, obliterating the patent processus vaginalis. The hernia sac was not divided. Success was defined as the visualization of closure of the patent processus vaginalis with no re-insufflation of the hernia sac. If re-insufflation was observed, a second suture was placed using the same technique. Fascia and skin wounds were closed with absorbable sutures and sterile braided adhesive strips, respectively. After observation of normal feeding, patients were discharged at the 4th–6th postoperative hour, if without pain. Oral or rectal paracetamol was utilized for pain relief. An outpatient follow-up visit was scheduled for the postoperative 7th day. Thereafter parents were advised to return if they had any concerns, particularly if there was bleeding, redness, swelling, or a mass in the incision areas.

Age at surgery and follow-up time were measured in months. Contralateral PPV was defined as a “cavernous” or “fissure”-like opening in the peritoneum at the level of the internal inguinal ring. Surgical time was measured in minutes and was noted as the time between skin prep and the completion of the wound dressing. The duration of time under anesthesia, again measured in minutes, was noted as the time of induction until the patient achieved a modified Aldrete score [13] of 9 or higher.

Statistical analysis was conducted using Google Sheets (Google LLC., Mountain View, California, United States), as well as the tools available at biyostat.com. We represented continuous data using the measures of means and standard deviations, while categorical data were presented as numerical values accompanied by their corresponding percentages.

The study received approval from the Ethics Committee of Maltepe University, Istanbul, Turkey (Approval No: 2024/11-08) and was conducted in compliance with the Declaration of Helsinki. Each parent or guardian provided informed consent for the surgical operation and for the usage of their children’s data in scientific research.

The primary outcome of the study was the rate of recurrence. The secondary outcomes were surgical time, time under anesthesia, presence of CPPV, and the rate of complications.

## 3. Results

An initial assessment of the patient files revealed that 1167 patients had undergone surgery for inguinal hernia during the study period. Of these, 809 (69.3%) underwent surgery using the PIRS technique. Long-term follow-up data were obtained for 715 patients (61.3%). A consent form was not available for one patient; therefore, 714 patients were included in the final analysis. Figure 1 shows the STROBE diagram of the study.

Characteristics and follow-up data of the patients included in the study are shown in Table 1. CPPV was observed in 124 patients (18.8%). A total of 714 patients underwent 892 PIRS procedures (490 right, 170 left, 54 bilateral, and 124 CPPV).

Eight complications were observed in six patients. In those undergoing unilateral PIRS, three patients had complications. Iliac vessel puncture was seen in two patients, one of which developed into a large hematoma, and in another patient, omental evisceration requiring reduction and closure of the fascia under general anesthesia was observed. Of three patients undergoing bilateral PIRS, two had bilateral and one had unilateral iliac vessel puncture. All cases of vessel puncture were managed by external compression.

During the average follow-up time of 6.9 ± 2.3 years, a recurrence of IH was observed in seven patients, accounting for 0.98% of the patients and 0.78% of the procedures. Of the seven patients with recurrences, four were observed in those undergoing unilateral PIRS and three in bilateral PIRS. The rates of recurrence per patient and per procedure were similar for those undergoing unilateral and bilateral PIRS. The average time from surgery to the development of recurrence was 3.2 ± 2.1 months (1.2–6 months) with no patient developing recurrence after the sixth postoperative month. Five patients with recurrence were male, and two were female. Both female patients had ipsilateral hernia with CPPV, and both recurrences were observed on the ipsilateral side. In the five male patients with recurrence, none had CPPV, and all had recurrence of the ipsilateral side.

Table 2 demonstrates the results of the study’s primary and secondary outcomes, as well as the comparison between patients undergoing unilateral versus bilateral PIRS.

As anticipated, the average surgical length was significantly longer in children who underwent bilateral IH repair (*p* < 0.01). Nevertheless, the duration of anesthesia was almost identical in unilateral or bilateral IH repair (*p* > 0.05).

## 4. Discussion

This study reports the long-term outcomes of children who underwent PIRS for IH. Our results demonstrate that PIRS is both safe and highly effective, with a low recurrence rate and minimal complications. In light of the paucity of data on the long-term follow-up of children who undergo laparoscopic IH repair and the concern of a potential increase in the recurrence rate when these techniques are employed, these results are significant. With an average follow-up duration of nearly 7 years (range 3–11 years), our findings offer a comprehensive evaluation of the long-term outcomes in children undergoing PIRS.

Many intra- and extra-corporeal techniques are available for minimally invasive repair of IH in children [8]. In order to perform high ligation with intracorporeal suturing, the majority of techniques require the use of two or three ports. This procedure can be both time-consuming and require some level of experience. On the other hand, PIRS uses a single port, making it possibly more cost-effective.

Surgical time and time under anesthesia are critical factors in any surgery, but more so when comparing laparoscopic vs. open techniques and when shorter times under anesthesia are preferable for children. In our group of patients, the average surgical time was 13.6 min and 19.9 min for unilateral and bilateral PIRS, while the time under anesthesia was 27.7 min and 33.9 min, respectively. Our results are shorter than the traditional repair times reported in the literature. In a comparison of open vs. laparoscopic repair in 1697 patients, Chong et al. [14] reported an incision time of 36 min for open and 25 min for laparoscopic unilateral IH repair and 56 min for open and 31 min for laparoscopic bilateral IH repair. In a systematic review that included 2196 patients, Petridou et al. [15] reported average operative times for laparoscopy assisted and laparoscopic repair of IH in children to be 21.49 ± 13.51 min vs. 29.73 ± 11.05 min for unilateral and 28.01 ± 15.08 min vs. 39.48 ± 16.35 for bilateral repair, respectively. When PIRS-specific studies are considered, the average surgical times are reported to be between 10 and 18 min for unilateral and 11 and 25 min for bilateral repair [16,17,18,19,20,21,22].

Various studies have reported the rate of CPPV to be between 20 and 30% [23]. Li et al. suggested that the “cavernous” type of PPV in patients <3 years of age with left initial hernia should undergo bilateral repair [24]. Zhao et al. reported that the number needed to treat to prevent one metachronous hernia was 21 for CPPV [25]. The presence of a CPPV and its management is a significant point of debate in laparoscopic IH repair. While detecting and repairing CPPV can prevent the development of a contralateral metachronous hernia, surgical intervention to a non-symptomatic condition is cause for serious debate. On one hand, the burden of surgery and anesthesia of a metachronous hernia can be avoided, while on the other hand, a potentially non-symptomatic finding with no traditional indication for surgery is treated, putting additional, and maybe unnecessary, risks to the child involved. We choose to repair all CPPVs when detected. Our data demonstrate that time under anesthesia only slightly increases by an average of six minutes, and the increase in time is not statistically significant (27.7 ± 12.9 vs. 33.9 ± 14.1, *p* > 0.05.)

Complications following IH surgery include blood-vessel injury, recurrence, formation of hydrocele, suture reaction, and scrotal swelling [20]. We observed eight complications in six patients (complication rate per patient: 0.84%, complication rate per procedure: 1.2%). In a comparison of 1827 patients undergoing open and 478 undergoing laparoscopic repair, Safa et al. reported complication rates of 5.1% and 6.9%, respectively, with the only statistically significant difference being a higher rate of wound infection in patients undergoing laparoscopy [26]. In a series of over 10,000 children, Gretch et al. reported complications in 0.7% of patients [27]. In a series comparing laparoscopic and open surgery amongst 1697 patients, with an average follow-up of nearly 4 years, Chong et al. reported complications in 0.43% for open and 1.11% for laparoscopic repair [14]. We observed a range of complications in our patients, primarily vessel puncture. Only one serious complication—omental evisceration, was observed.

Recurrence is a crucial topic of discussion in IH repair. The recurrence rate for laparoscopic techniques ranges from 0.3% to 11% [26]. Nevertheless, the problem with these data lies in the substantial variation in follow-up times across different studies. Reported risk factors for recurrence include prematurity, emergency treatments, and repairs performed in neonates [26]. Chong et al. reported a recurrence rate of 0.71% and a recurrence rate per 1000 person years of 2.2, with 75% of recurrences occurring within 6 months [14]. We observed a recurrence rate of 0.98% per patient and 0.78% per procedure. These results are similar to the findings reported in recent systematic reviews and meta-analyses. In a comparison of laparoscopic and open IH repairs, Zhao et al. compared data from 13 randomized controlled trials (RCTs) involving 1207 patients and reported no difference between recurrence rates when open and laparoscopic IH repair was compared [1]. The authors noted that long-term complications, such as recurrence, may be underestimated due to differing follow-up times. Their systematic review referenced Grosfeld et al.’s report from 1991 that found that recurrences of IH in children occur within 6 months in 50%, within 2 years in 76%, and within 5 years in 96% of children [28]. In an analysis of three RCTs and four observational clinical studies compassing 1543 patients undergoing laparoscopic and 657 patients undergoing open IH repair, Yang et al. reported similar recurrence rates, 1.04% for laparoscopic repair vs. 1.98% for open repair [29]. The average follow-up time for patients undergoing laparoscopic repair was reported to range between 3 to 120 months. The longest follow-up included in the analysis was Endo et al.’s study [30], which reported a follow-up time between 1 and 10 years, although no mean or median was provided. Amongst the remaining studies, the average follow-up was 24 months. Furthermore, in a meta-analysis comparing open vs. laparoscopic IH repair, Kantor et al. reported recurrence to range between 0 and 6.3% for open and 0 and 5.7% for laparoscopic surgery, with the variability attributed to differing techniques [31]. Moreover, the follow-up times reported were all <2 years. In lieu of these conclusions, the follow-up averaging nearly 7 years (range 3–11) in our cohort is significant. However, contrary to Zhao et al.’s findings [1], all the recurrences in our cohort occurred within the first 6 months of surgery. No patients were observed as having testicular atrophy, as reported by parents. Long-term follow-up, as seen in our study with an average follow-up period of 6.9 years, is crucial for assessing the durability of surgical interventions in pediatric populations. Studies by Li et al. [24] and others have emphasized the importance of long-term data to validate the efficacy of laparoscopic techniques, particularly in preventing recurrences and managing complications over time. Our findings contribute to this growing body of evidence, underscoring the reliability of PIRS for long-term IH outcomes. Notably, the low recurrence rates and minimal complications observed over an extended follow-up period highlight the durability and effectiveness of the PIRS technique.

Despite the promising results, our study has some limitations, including its retrospective design. Although we report one of the longest follow-up cohorts in the literature, our patient and procedure numbers are low. The study, by design, relied on a telephone survey to evaluate long-term follow-up. Future research aiming to determine if laparoscopic IH repair should replace open surgery as the gold standard should focus on prospective, multicenter studies with large sample sizes and standardized protocols to further validate the benefits of PIRS or other minimally invasive techniques. Comparative studies between PIRS and other laparoscopic techniques, as well as open surgery, will be invaluable for establishing the most effective approach for pediatric IH repair.

## 5. Conclusions

To conclude, our study offers substantial evidence regarding the long-term results of PIRS for pediatric IH repair. It demonstrates the safety and efficacy of the procedure, as evidenced by its minimal complications and low recurrence rates over an average follow-up period of nearly 7 years. This research addresses critical knowledge voids concerning the durability of minimally invasive techniques in pediatric IH repair. In order to further validate these findings and establish PIRS as the preferred approach, prospective, multicenter studies with larger sample sizes and standardized protocols are necessary.

## Figures and Tables

**Table 1:** Gender, age at surgery, side of presentation of inguinal hernia, and follow-up data of patients included in the study.

Characteristic	Results
Gender, n (%)	
Male	520 (72.8%)
Female	194 (27.2%)
Age at Surgery (months)	
Median	20.0 ± 19.8
Mean	20.0
Range	0.1–121
Side at Presentation, n (%)	
Right	490 (68.6%)
Left	170 (23.8%)
Bilateral	54 (7.6%)
Follow-up Time (years)	
Median	6.9 ± 2.3
Mean	6.8
Range	3–11.1

**Table 2:** Primary and secondary outcome results, as well as their comparison between patients undergoing unilateral vs. bilateral PIRS.

Type of Outcome	Outcome	All Patients	Unilateral PIRS	Bilateral PIRS	*p*
Primary	Number of Recurrences, n	7	4	3	>0.05
	Rate of Recurrence per patient, %	0.98%	0.61%	1.69%	>0.05
	Rate of Recurrence per procedure, %	0.78%	0.61%	1.29%	
Secondary	Presence of CPPV, n (%)	124 (18.8%)		N/A	
	Surgical Time (minutes), median ± SD	14.7 ± 5.6	13.6 ± 5.4	19.9 ± 3.0	<0.001
	Anesthesia time (minutes), median ± SD	28.8 ± 13.3	27.7 ± 12.9	33.9 ± 14.1	>0.05
	Complications per patient, n (%)	6 (0.84%)	3 (0.45%)	3 (1.69%)	>0.05
	Complications per procedure, n (%)	8 (1.2%)	3 (0.45%)	5 (2.16%)	<0.05

## Data Availability

The data presented in this study are available on request from the corresponding author.
